# Region-specific diversification of the highly virulent serotype 1 *Streptococcus pneumoniae*

**DOI:** 10.1099/mgen.0.000027

**Published:** 2015-08-11

**Authors:** Jennifer E. Cornick, Chrispin Chaguza, Simon R. Harris, Feyruz Yalcin, Madikay Senghore, Anmol M. Kiran, Shanil Govindpershad, Sani Ousmane, Mignon Du Plessis, Gerd Pluschke, Chinelo Ebruke, Lesley McGee, Beutel Sigaùque, Jean-Marc Collard, Martin Antonio, Anne von Gottberg, Neil French, Keith P. Klugman, Robert S. Heyderman, Stephen D. Bentley, Dean B. Everett, for the PAGe Consortium

**Affiliations:** ^1^​ The Malawi-Liverpool-Wellcome Trust Clinical Research Programme, University of Malawi College of Medicine, Blantyre, Malawi; ^2^​ University of Liverpool, Institute of Infection and Global Health, Liverpool, UK; ^3^​ The Wellcome Trust Sanger Institute, Wellcome Trust Genome Campus, Hinxton, Cambridge, UK; ^4^​ Medical Research Council, Banjul, The Gambia; ^5^​ Division of Translational and Systems Medicine, Microbiology and Infection Unit, The University of Warwick, Coventry, UK; ^6^​ National Institute for Communicable Diseases, Division of the National Health Laboratory Service; and School of Pathology, Faculty of Health Sciences, University of the Witwatersrand, Johannesburg, South Africa; ^7^​ Centre de Recherche Médicale et Sanitaire, Niamey, Niger; ^8^​ Swiss Tropical and Public Health Institute, Basel, Switzerland; ^9^​ Faculty of Infectious and Tropical Diseases, London School of Hygiene and Tropical Medicine, London, UK; ^10^​ Centers for Disease Control and Prevention, Atlanta, GA, USA; ^11^​ Centro de Investigação em Saúde da Manhiça, Maputo, Mozambique; ^12^​ Hubert Department of Global Health, Rollins School of Public Health, Emory University, Atlanta, GA, USA

**Keywords:** Pneumococcal Disease, Genomics, Phylogeny, Africa, Recombination, Antibiotic Resistance, PAGe

## Abstract

Serotype 1 *Streptococcus pneumoniae* is a leading cause of invasive pneumococcal disease (IPD) worldwide, with the highest burden in developing countries. We report the whole-genome sequencing analysis of 448 serotype 1 isolates from 27 countries worldwide (including 11 in Africa). The global serotype 1 population shows a strong phylogeographic structure at the continental level, and within Africa there is further region-specific structure. Our results demonstrate that region-specific diversification within Africa has been driven by limited cross-region transfer events, genetic recombination and antimicrobial selective pressure. Clonal replacement of the dominant serotype 1 clones circulating within regions is uncommon; however, here we report on the accessory gene content that has contributed to a rare clonal replacement event of ST3081 with ST618 as the dominant cause of IPD in the Gambia.

## Data Summary

1. Supplementary Tables S3 & S5 have been deposited in FigShare: DOI – 10.6084/m9.figshare.1472901 (URL – http://dx.doi.org/10.6084/m9.figshare.1472901).
2. Study genomes have been deposited in GenBank; accession numbers are detailed in Supplementary Table S6 which has been deposited in FigShare: DOI – 10.6084/m9.figshare.1472901 (URL – http://dx.doi.org/10.6084/m9.figshare.1472901).

**Impact Statement**

*Streptococcus pneumonia* serotype 1 is a leading cause of pneumococcal pneumonia and meningitis globally, with the highest burden of disease in the developing world. In this study we sequenced a global collection of *S. pneumoniae* serotype 1 isolates and show that serotype 1 in Africa is genetically distinct from serotype 1 in the developed world. Within Africa, serotype 1 has disseminated and diversified between countries in response to region-specific selective pressures. Recombination and antibiotic usage have contributed to this diversification. Of interest, serotype 1 is subject to the same level of recombination as other serotypes commonly associated with nasopharyngeal carriage. This contradicts the view that short duration of carriage limits the opportunity for serotype 1 to recombine and acquire antibiotic resistance mechanisms. We demonstrate that long-range transmission of serotype 1 is rare, with locally circulating clones in countries remaining stable with little impact from imported clones. In a rare example of clone replacement, the serotype 1 clone ST3081 replaced ST618 as the dominant cause of invasive pneumococcal disease in the Gambia in 2006. We report on the virulence factors unique to ST3081, which have likely driven this replacement. This is the largest reported sequencing collection of a single *S. pneumoniae* serotype to date. Our analysis shows how country-specific selective pressures have driven the evolution and diversification of this important pathogen within Africa.

## Introduction

*Streptococcus pneumoniae* is a commensal bacterium commonly isolated from the human nasopharynx (Turner *et al.*, 2012). It is a major cause of morbidity and mortality worldwide, manifesting as a range of clinical infections, from sinusitis and acute otitis media to meningitis, septicaemia and pneumonia, with the highest disease burden in developing countries (O'Brien *et al.*, 2009). *S. pneumoniae* is conventionally subdivided into more than 90 different serotypes (Bentley *et al.*, 2006), which have differing disease associations (Hausdorff *et al.*, 2005; Yildirim *et al.*, 2010). For a century, serotype 1 has ranked among the most prevalent pneumococcal serotypes causing invasive pneumococcal disease (IPD) worldwide (Harboe *et al.*, 2010). In Africa, serotype 1 is the second most common cause of IPD (proportion of IPD: 11.7 %) after serotype 14 (Johnson *et al.*, 2010).

Serotype 1 has distinct characteristics compared with other pneumococcal serotypes; it shows even distribution of incidence across all age ranges, is linked to outbreaks in closed communities and is associated with epidemic outbreaks in West Africa (Antonio *et al.*, 2008; Dagan *et al.*, 2000; Ritchie *et al.*, 2012). Furthermore, serotype 1 is rarely associated with antimicrobial resistance (Brueggemann *et al.*, 2003; Hausdorff *et al.*, 2005). Short duration and low densities of serotype 1 during colonization may explain low resistance levels because recombination with other streptococci in the nasopharynx is a major source of resistance in pneumococci (Brueggemann & Spratt, 2003; Williams *et al.*, 2012).

The high burden of disease caused by serotype 1 was the impetus for clinical trials of the nine-valent conjugate vaccine (PCV9) in the Gambia and South Africa, the first conjugate vaccine to incorporate serotype 1 capsular polysaccharide (CPS) (Cutts *et al.*, 2005; Klugman *et al.*, 2003). PCV10 and PCV13 are currently being rolled out across Africa. Both incorporate serotype 1 CPS (Klugman *et al.*, 2008); however, comprehensive impact evaluation data for these vaccines are not yet available (Blumental *et al.*, 2015).

Genetic characterization of a global collection of 166 serotype 1 pneumococci using MLST identified three lineages each associated with different regions of the world, with lineage B associated with Africa (Brueggemann & Spratt, 2003). Within regions there was further suggestion of phylogeographic structure but the sample size and low resolution of MLST limited the conclusions that could be made. In recent years, whole genome sequencing has emerged as a practical method for studying bacterial genetics across large numbers of samples, allowing reconstruction of high-resolution phylogenies and correlation of complete gene catalogues with phenotypic and clinical data (Croucher *et al.*, 2011; Harris *et al.*, 2010; Köser *et al.*, 2012).

Here we present a whole genome phylogeny of 448 serotype 1 pneumococcal isolates, recovered from 27 countries worldwide (including 11 in Africa) in order to investigate the genetic diversity of this important pathogenic serotype and relate this to clinical phenotype. We also investigate the evolutionary mechanisms and specific selective pressures that have driven the diversification of serotype 1 within Africa.

## Methods

### Whole genome sequencing

*S. pneumoniae* was cultured and genomic DNA extractions were performed as described elsewhere (Everett *et al.*, 2011). Multiplex DNA sequencing using the Illumina Genome Analyser GAII (Illumina) was performed, as previously described (Harris *et al.*, 2010). The sequence reads generated were deposited in the European Nucleotide Archive (ENA) (http://www.ebi.ac.uk/ena/) under study number ERP000156, a full list of accession numbers is available in Table S1 (available in the online Supplementary Material). All isolates had previously tested positive as serotype 1 pneumococci by PCR using a standard protocol (Pai *et al.*, 2005). To confirm all of the study isolates were serotype 1 we also employed an additional *in silico* serotyping method, whereby the sequence reads were redundantly aligned against the concatenated sequences of 94 pneumococcal CPS loci using BWA (Li & Durbin, 2009). The CPS locus with the highest proportion of its length covered by mapped reads was taken as the serotype (Croucher *et al.*, 2011). We observed 100 % concordance between the PCR and *in silico* serotyping results; all of the study isolates exhibited the highest mapping to the serotype 1 CPS locus. The seven MLST loci were extracted from the assembled sequence reads and compared with the MLST.net pneumococcal database using the short read sequence typing (SRST) tool (Inouye *et al.*, 2012).

### Phylogeny reconstruction

Sequence reads were mapped against serotype 1 *S. pneumoniae* P1041 (accession number: FQ312030) using smalt (http://www.sanger.ac.uk/resources/software/smalt), giving, on average, 185 ×  depth of coverage for more than 94.4 % of the reference genome (Fig S1). SNPs were identified as described by Harris *et al.* (2010). A phylogenetic tree was reconstructed for all SNP sites in the genomes using RAxML v.7.0.4 (Stamatakis, 2006). A General Time-Reversible (GTR) model with gamma correction for among-site variation was used with 10 starting trees. To assess support for nodes, 100 random bootstrap replicates were performed. Recombinant sites in the lineage B isolates were identified as previously described and the phylogeny was reconstructed for all SNP sites independent of recombinant blocks (Croucher *et al.*, 2011).

### Nucleotide substitution rate

Rates of single nucleotide substitution for the five lineage B clades, which featured African isolates (clades i, ii, iii, v and vi) were calculated with Bayesian Evolutionary Analysis by Sampling Trees (beast), using 2 000 000 Markov Chain Monte Carlo (MCMC) iterations sampled every 1000 steps (Drummond *et al.*, 2012). The nucleotide substitution rate between clades was compared using the Kruskal–Wallis test. Rates of recombination for each clade were calculated as previously described (Croucher *et al.*, 2011) and compared using the Kruskal–Wallis Test.

### Genome assembly and annotation

Genomes for the lineage B isolates were *de novo* assembled using a pipeline, which iteratively ran Velvet (Zerbino & Birney, 2008) (with k-mer size ranging between 60 and 90 % of the read length), smalt and sspace (Boetzer *et al.*, 2011). This pipeline gave on average a total length of 2 063 265 bp, with average contig length of 19 660 bp and average N50 of 9733 bp. Assembly statistics are summarized in supplementary Table S1. *De novo* gene prediction was performed using Glimmer (Delcher *et al.*, 1999) and annotation was transferred using prokka (Seemann, 2014).

### Antimicrobial resistance analysis

Individual gene trees for *folA* and *folP* were reconstructed with RAxML v.7.0.4 (Stamatakis, 2006) using a GTR model with GAMMA correction using 100 bootstraps. Display and manipulation of the single gene phylogenetic trees was performed using the online interactive tree of life (Letunic & Bork, 2011). On the basis of the prediction of recombination in isolates from the four main study sites (Malawi, The Gambia, South Africa and Niger) for which complete antimicrobial resistance data were available, isolates undergoing *folA* and *folP* recombination and their phenotypic resistance to co-trimoxazole were compared with strains with no recombination events detected at these sites. A Kruskall–Wallis test was used to estimate the statistical difference between the two groups.

### Core genome analysis (lineage B)

Annotated genes were translated to protein sequences and assigned to orthologous gene ‘clusters’ using OrthoMCL, with blastp E-value cut-off 1e^− 5^ and inflation index 1.5 (Li *et al.*, 2003). A stringent quality control process was applied to the genome assemblies, to ensure that poor assembly of small genomic regions did not result in an underestimate of the core genome size (Table S1). The resulting orthologous clusters were organized into a matrix of genome content using bespoke Perl scripts, with orthologues from the same genome arranged in columns and rows identifying annotated orthologues of similar function. A blank (‘-’) was inserted where an orthologue was absent. Genomes (the matrix columns) were randomly sampled in an arithmetic progression fashion: *S*_*N*_* = N/2*[*2a+(N − 1)d*]. The number of random sampling events, S_N_, was established using the least number of genome(s) under consideration, *a* (i.e. 1 genome in this study), the total number of genomes under consideration (i.e. the dataset size), *N*, and the common difference of successive genomes, *d*, (i.e. 1) to be used during sampling. During random sampling, the total number of genomes, *N*, was initialized as one genome for the first event, and increased by one unit for each of the subsequent events, until it was equivalent to the total number of genomes in the dataset in the final event. The random genomes were only sampled once during each event and the total number of orthologues shared by all genomes was counted once for each cluster, thereby excluding paralogous counts. This was iterated 100 times resulting in 100 input orders for each event; the arithmetic mean core genome size was computed for each event to enable the mean core genome size to be related to the number of genomes sampled (Fig. S2). Comparisons of core genomes from different datasets were achieved using Perl scripts (https://github.com/fy2/pneumoscript).

## Results

### Global population structure of serotype 1

**Fig. 1.**
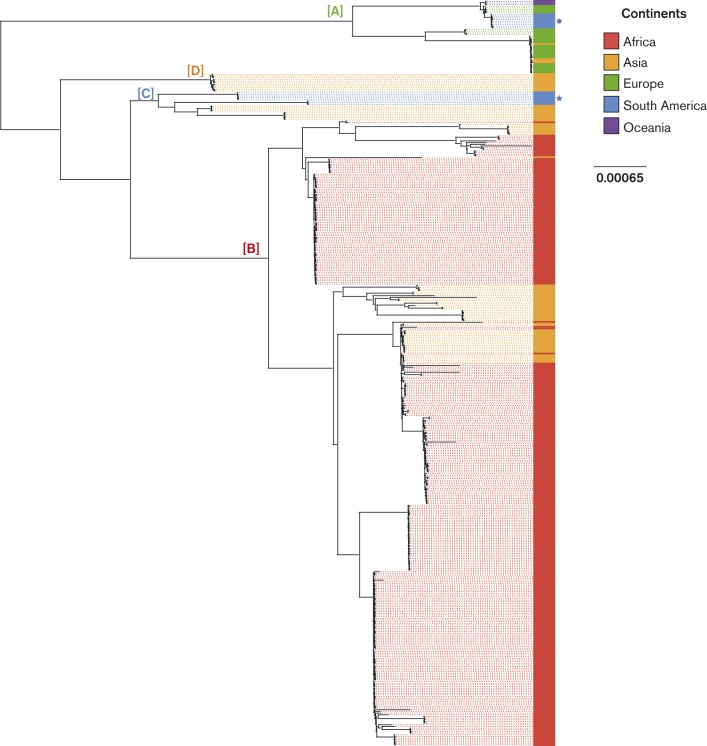
Phylogeography of serotype 1 *S. pneumoniae* isolates. Maximum-likelihood phylogenetic tree based on the whole genome SNPs of serotype 1 isolates annotated with country of origin. The colour of each isolate indicates the continent of origin: red, Africa; orange, Asia; green, Europe; blue, South America (the Brazilian group is highlighted by a circle, the Argentinian and Peruvian isolates are highlighted by a star); purple, Oceania. Specific lineages referred to in the text are labelled: A (lineage A, Europe, South America & Oceania), B (lineage B, Africa), C, (lineage C, South America), D (lineage D, Asia). Note that Asian isolates are present in all of the lineages.

Whole genome sequencing was performed for 448 serotype 1 isolates collected from 27 countries between 1994 and 2009 (see supplementary material for sampling strategy, Fig. S3, Table S2, Table S3). Short reads for each isolate were mapped against the genome of serotype 1 *S. pneumoniae* P1041 and SNPs were identified. A maximum-likelihood phylogeny based on 58 718 SNPs is presented in Fig. 1. The study isolates grouped into four lineages (A–D). Lineages A to C have been previously reported based on MLST (Brueggemann & Spratt, 2003); here we report a previously unrecognized clade (lineage D), composed of Asian isolates. We observed a striking continental clustering, indicating significant geographical structure in the global population. The African isolates all grouped within a single lineage (lineage B). The European isolates all fell within lineage A. All isolates from Oceania fell within a distinct lineage A subclade. In contrast, isolates from Asia were found in all four lineages, suggesting multiple intercontinental transfer events. The South American isolates grouped into two lineages; isolates from Peru and Argentina formed distinct lineage C subclades and isolates from Brazil grouped within lineage A. Despite the inclusion of a low number of isolates from outside of Africa, four continents (Asia, Europe, South America and Oceania) were represented in lineage A, suggesting this lineage is subject to a relatively greater frequency of intercontinental transfer events.

### Phylogeography of serotype 1 within Africa

**Fig. 2.**
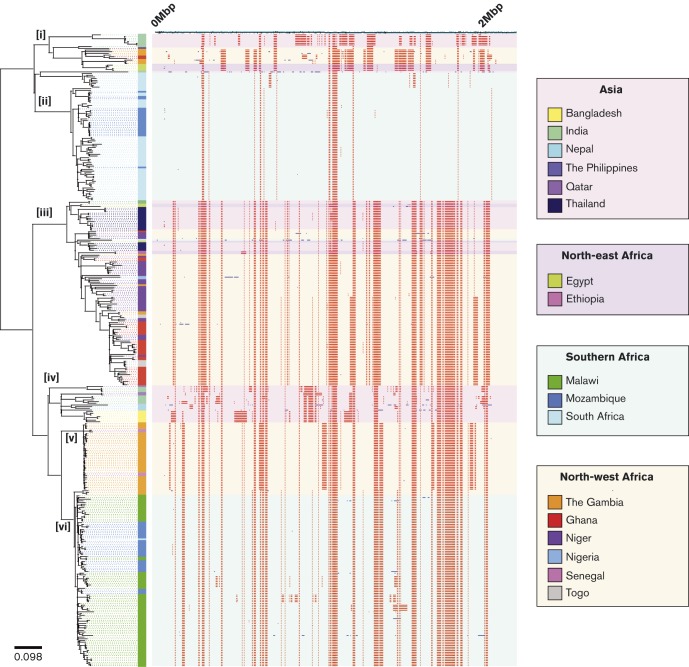
Reconstructed maximum-likelihood phylogeny of serotype 1, lineage B isolates. The phylogeny is solely reconstructed using SNPs outside of recombinant blocks. The scale bar represents substitutions per SNP. The colour of each isolate indicates the country of origin. The panel next to the phylogeny shows the genomic locations of putative recombination events detected in each terminal taxon. Red blocks indicate recombination events that have occurred in multiple isolates. Blue blocks indicate recombination events that have occurred in a single isolate. The background colour of the panel indicates the region of origin.

The lineage B phylogeny was reconstructed with variation due to recombination removed (Croucher *et al.*, 2015) (Fig. 2). The phylogeny consisted of six major clades (labelled i–vi) and showed a high level of geographical structure. The Malawi isolates formed a single clade along with isolates from Mozambique (clade vi), indicating serotype 1 pneumococci remained genetically stable in Malawi over the sampling period with no detectable impact from imported clones. The South Africa isolates (with two exceptions) formed a single clade, ii. Malawi and South Africa are in relatively close geographical proximity, yet clades vi and ii are phylogenetically distant, suggesting genotypic stability of serotype 1 in these countries and limited inter-country transfer. A South African isolate grouped within clade iv; subsequent research revealed this isolate was recovered from a Mozambican in South Africa. The Mozambique isolates fell in two clades, 30/51 (59 %) clustered with the Malawi isolates (clade vi) and 21 (41 %) clustered with the South Africa isolates (clade ii). This indicates multiple inter-country transfers between Mozambique and bordering countries, Malawi and South Africa. Civil war in Mozambique saw a mass influx of 1.7 million refugees into bordering countries, the post-war return of refugees to Mozambique may have led to the importation of serotype 1 clones which became dominant within Mozambique (United Nations High Commissioner for Refugees, 2000).

The North African isolates showed clear separation from the southern African isolates (Fig. S4). The majority (61 %, 88/145) lay within the second most diverse clade, iii (based on average terminal branch lengths; Fig. S5), which also contained isolates from Asia. Despite the high number of countries represented in clade iii there was a relatively high level of country-specific clustering, implying that inter-country transfer events were infrequent. However, a single South African isolate grouped within clade iii, inferring a cross-region transfer event.

The Gambia isolates were an anomaly, only a subset (6 %, 3/50) grouped within clade iii with the other West African isolates. The majority clustered in clades i and v. The Gambia isolates in clade i (14 %, 7/50) and v (72 %, 36/50) belonged to ST618 and ST3081, respectively (Fig. S6). In the Gambia, ST618 previously dominated, causing >70 % of serotype 1 IPD from 1995 to 2006 (Antonio *et al.*, 2008). ST3081, first detected in the Gambia in 2006, has replaced ST618 as the dominant cause of IPD. ST3081 has only been previously reported in Oman in 2004 (MLST.net). Consistent with the expansion of a newly emergent clone, clade v was the least diverse within the phylogeny (Fig. S5). A further three Gambia isolates (6 %) grouped within clade vi, separated from the Malawian and Mozambique subclade by a long branch length, suggesting a historical cross-region transfer event. Clade v consisted solely of isolates from Asia and was excluded from subsequent analyses.

### Recombination and resistance

**Fig. 3.**
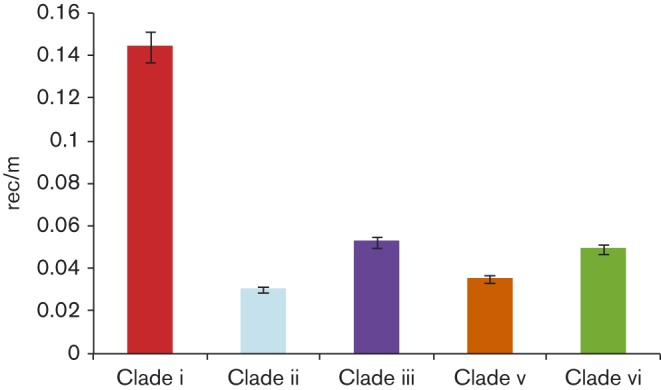
Recombination dynamics of serotype 1 pneumococci. The mean rec/m (number of homologous recombination events/ number of SNPs introduced through spontaneous mutation) for the lineage B African clades. Error bars represent the 95 % confidence interval.

We next investigated the evolutionary mechanisms that contributed to the diversification of serotype 1, lineage B in Africa. Consistent with the substitution rate reported elsewhere, (Croucher *et al.*, 2011, 2013) the mean estimated substitution rates of the lineage B African clades ranged from 1.714 × 10^− 6^ to 5.980 × 10^− 6^ per site per year (*P* = 4.6 × 10^− 9^). Of 38 187 SNPs identified, 30 597 (79.9 %) were introduced by 650 recombination events, ranging in size from 3 bp to 82 054 bp (Fig. S7). We found a significant difference in the rec/m (ratio of homologous recombination events to point mutations) between clades (*P* = 3.2 × 10^− 4^), which ranged from 0.03 in clade ii to 0.14 in clade i (Fig. 3). This is the first demonstration of varying recombination rates within a single pneumococcal lineage.

**Fig. 4.**
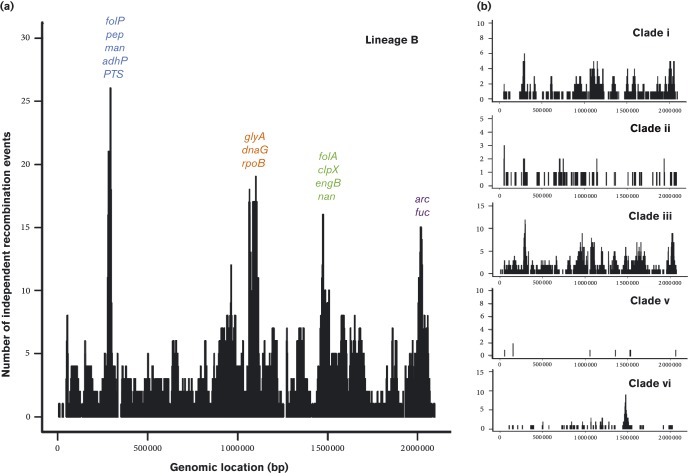
(a). Genomic regions recombination in the 376 lineage B isolates. The genes subject to the highest number of independent recombination events are named. (b). Genomic regions under recombination within the five lineage B Africa clades. *folP*, dihydropteroate synthase; *pep*, aminopeptidase; *man*, mannose PTS system component; *adhP*, alcholol dehydrogenase; *PTS*; phosphotransferase system protein; *glyA*, serine hydroxymethyltransferase; *dnaG*, DNA primase; *rpoB*, RNA polymerase beta subunit; *folA*, dihyrdofolate reductase; *clpX*, CLP protease; *engB*, STP binding protein; *nan*, neuraminidase; *arc*, arginine deiminase; *fuc*, fuculose kinase.

The majority of recombination events detected were on branches between clades (ancestral recombination) (426/650, 66 %) (Fig. 2). Analysis of recombination events on branches within clades (recent recombination) showed a number of genes recombined multiple times in some clades, but did not undergo recombination in others. These recombination events may have been driven by clade-specific selective pressures (Table S4) (Fig. 4). Unique to clade iii isolates from the Gambia, Ghana, Niger and Togo (and a subset of Asian isolates), were recombination events involving *pbp1a*, *pbp1b* and *pbp2a*, allelic variants of which confer beta-lactam resistance (Cornick & Bentley, 2012). Likewise, *gyrA* and *parC*, allelic variants of which confer fluoroquinolone resistance, were only subject to recombination in Malawi isolates (clade vi). Insufficient antimicrobial resistance data were available to correlate recombination to resistance; however, it is probable that recombination in these areas reflects high antimicrobial consumption.

**Fig. 5.**
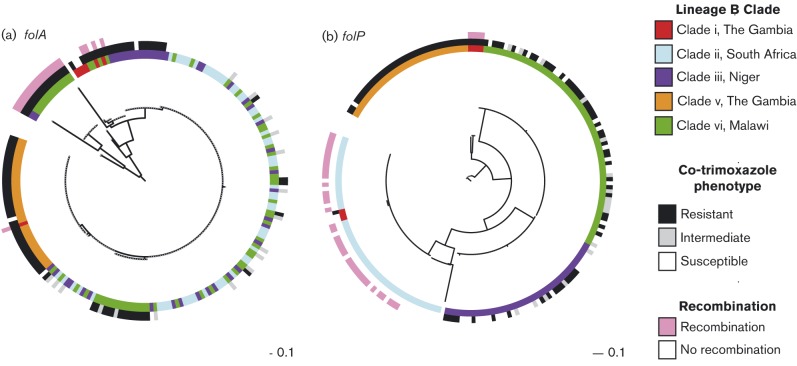
Association between clade-specific recombination events involving *folA* and *folP* and co-trimoxazole resistance. (a) The centre phylogeny is based on the SNP differences between *folA* from 210 lineage B isolates. (b) The centre phylogeny is based on the SNP differences between *folP* from the same dataset. The inner circles represent the lineage B clades from which the genes were isolated. The middle circles are coloured according to the co-trimoxazole resistance phenotype. The outer ring indicates if *folA* or *folP* was involved in a clade-specific recombination event. The scale bar represents substitutions per SNP.

The gene subject to the highest number of unique recombination events in clades i and iii was *folP* (dihydropteroate synthase); *folA* (dihydrofolate reductase) was also subject to multiple recombination events in clades iii and iv. Allelic variants of these two genes confer resistance to co-trimoxazole and sulfadoxine/pyrimethamine (SP) (Cornick *et al.*, 2014) (Fig. 5, Table S5). We investigated if there was an association between recent recombination and antimicrobial resistance for the 210 isolates from the four main study sites where resistance data were available; (the Gambia *n* = 43, Malawi *n* = 64, Niger *n* = 31, South Africa *n* = 58). The isolates that underwent recent *folA* recombination (10 %, 25/210) were phenotypically more resistant than those that had not (average MIC 4.0 vs 2.1 mg ml^− 1^, *P* = 1.9 × 10^− 4^), in contrast to a previous report (Chewapreecha *et al.*, 2014). However, isolates that had undergone a recombination at *folP* (33 %, 40/210) were phenotypically less resistant than those that had not (average MIC 0.7 vs 2.6 mg ml^− 1^, *P* = 9.5 × 10^− 15^).

Recent recombination events in *folA* were unique to a subset of the Malawian isolates and a single isolate from Niger. All of the Malawi isolates were co-trimoxazole resistant; recent recombination had introduced the resistant *folA* genotype (Ile-100-Leu of DHFR). All of the Malawi isolates also possessed the *folP* resistance genotype (a 1–2 aa insertion in DHPS). The *folP* phylogeny (Fig. 5) showed that the *folP* sequences clustered based on country of isolation, suggesting that the *folP* resistance genotype was introduced through a historical recombination event or spontaneous mutation and disseminated within the population. SP was extensively used as a first-line treatment for uncomplicated malaria in Malawi from 1993 to 2007. In 2005, co-trimoxazole preventative therapy (CPT) for individuals with HIV became national policy. This represents a potentially strong selective pressure for pneumococci to acquire and maintain co-trimoxazole resistance. Recent recombination events at *folP* were unique to a subset of South African isolates and four Gambian isolates. Despite extensive CPT use and evidence of recent *folP* recombination in the South African isolates, no resistance was observed in these isolates. South Africa, however, has a low incidence of malaria and therefore low SP consumption. The recent recombination events in *folP* in the South African isolates may be a result of the introduction of CPT placing a ‘new’ selective pressure and in time a recombination event may introduce the resistance genotype.

### Resistance due to mobile genetic elements

**Fig. 6.**
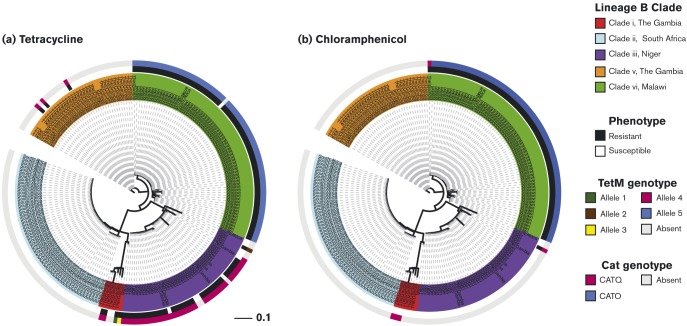
Distribution of the antimicrobial resistance proteins Tet and Cat in serotype 1 pneumococci. The centre phylogeny is based on the whole genome SNP differences between 210 lineage B isolates, the inner rings are annotated with the country of origin and coloured according to the lineage B clade which the isolate belongs to. (a) The middle ring is coloured according to the tetracycline resistance phenotype, the outer ring according to the absence/presence of Tet. (b) The middle ring is coloured according to the chloramphenicol resistance phenotype, the outer ring according to the absence/presence of Cat. The scale bar represents substitutions per SNP.

Amongst the subset of 210 isolates we found that 49 % (102) were tetracycline-resistant and 31 % (65) were chloramphenicol-resistant (Table S5). The presence of *tetM* and *cat*, which confer tetracycline and chloramphenicol resistance, respectively, was assessed. *TetM* was identified in 53 % (112) and *cat* in 33 % (70) of isolates (Fig. 6). Two allelic variants of TetM dominated in the dataset, one associated with north-west Africa and the other southern Africa, both alleles were associated with a MIC of >8 μg ml^− 1^. All but one of the Malawi (clade vi) isolates harboured TetM; conversely none of the South Africa (clade ii) isolates encoded TetM. TetM was identified in all three of the north-west African clades, including 84 %, (26/31) of the Niger isolates and four Gambia isolates (9 %, 4/43). Allelic variants of Cat were also associated with different African regions. CatO was only identified in southern Africa; consistent with high phenotypic resistance, all of the Malawi isolates encoded CatO. The CatQ protein was unique to north-west Africa; however, it was only identified in three Gambia isolates (clade i), none of which displayed phenotypic resistance, and a single resistant Niger isolate. Tetracycline and chloramphenicol are readily available across Africa and it is unclear why these resistance mechanisms are only present in specific countries. The findings contrast with other studies, that report low levels of antimicrobial resistance in serotype 1 (Williams *et al.*, 2012).

### Region- and lineage-specific gene content may explain differences in disease phenotype

We defined the core and accessory serotype 1 genome for the six African lineage B clades. We identified 2305 orthologous gene clusters in the dataset. Fifty-nine per cent (59 %) (1358 core genes) were present in all of the study isolates. The remaining 842 (37 %) genes were present in two or more isolates (accessory genes), whilst 105 genes (5 %) were unique to a single isolate.

**Fig. 7.**
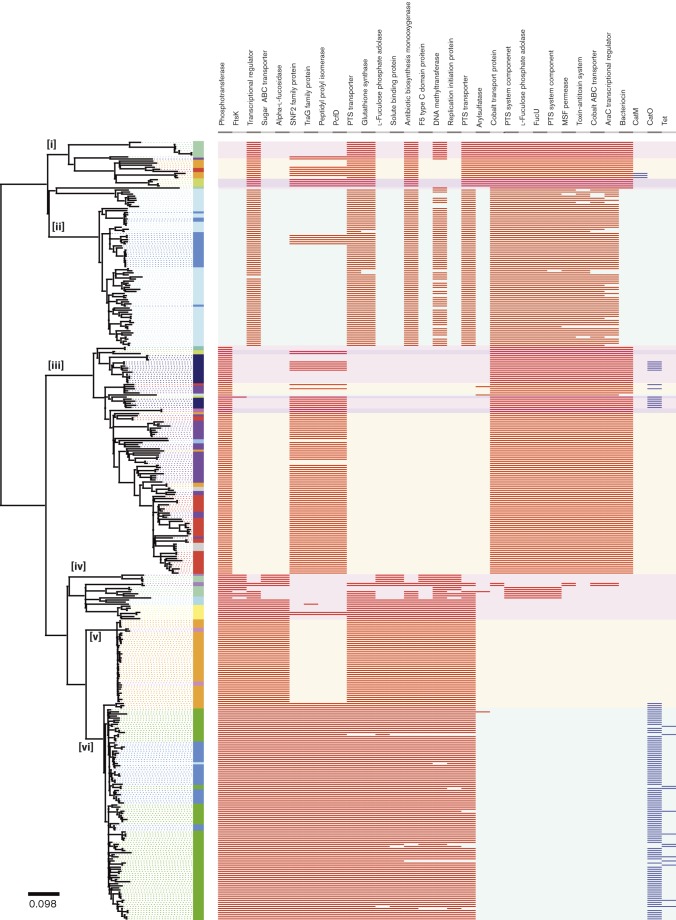
Reconstructed maximum-likelihood phylogeny of serotype 1, lineage B isolates. The phylogeny is solely reconstructed using SNPs outside of recombinant blocks. The scale bar represents substitutions per SNP. The colour of each isolate indicates the country of origin. The panel next to the phylogeny shows the absence/presence of accessory genes in each isolate. Red blocks indicate an accessory gene is present (blue blocks indicate an antimicrobial resistant accessory gene is present). Putative or hypothetical accessory genes are not included in this figure.

We identified 53 accessory genes that were absent in at least one clade but present in 100 % of isolates in at least one clade (Fig. 7, Table S6). Unique to the predominantly North African clades (i and iii) was a bacteriocin 972 family protein. This protein has been shown to contribute to the infectivity of *Streptococcus iniae* (Li *et al.*, 2014). A peptidylprolyl isomerase (PpiA) protein was present in the Malawi and Mozambique isolates in clade vi and a subset of North African isolates (clade iii). A homologue of PpiA reduces phagocytosis in *Streptococcus mutans* (Iyer *et al.*, 2001). Three genes from a phosphotransferase system (PTS) were present in all of the isolates in the North African clades (i and ii). PTS plays a key role in pneumococcal colonization (Mukouhara *et al.*, 2011). The distribution of these accessory genes may reflect host-specific selective pressures between regions and explain differences in disease severity between these regions.

**Fig. 8.**
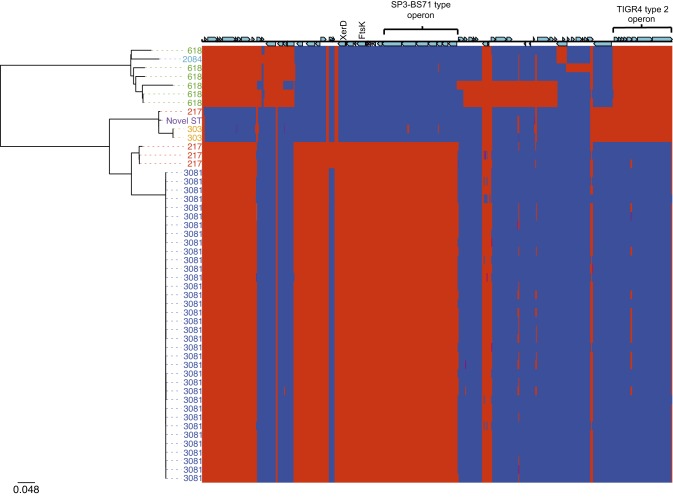
Phylogeny of ST618 and ST3081 serotype 1 *S. pneumoniae* isolates recovered from the Gambia. Maximum-likelihood phylogenetic tree based on the whole genome SNPs of serotype 1 isolates annotated with ST. The scale bar represents substitutions per SNP. The colour of each isolate indicates its ST: blue, ST3081; red, ST217; purple, novel ST; orange, ST303; green, ST618; turquoise, ST2084. The panel next to the phylogeny shows the presence (red) or absence (blue) of accessory genes for each of the study isolates.

As discussed earlier, the lineage B phylogeny (Fig. 2) indicates that there were two distinct, genetically unrelated groups of isolates from the Gambia, ST618 and ST3081. In 2006, ST3081 replaced ST618 as the dominant cause of IPD in the Gambia. MLST does not have the resolution to distinguish the genes exclusive to ST3081, which have driven sequence type (ST) replacement. To look at large accessory elements, rather than absence/presence of individual genes, we took a semi-manual approach to the creation of the Gambia accessory genome (see Methods) (Fig. 8). Accessory regions present in all ST3081 isolates but absent in ST618 isolates included a 6.2 kb phage element, encoding an integrase site-specific recombinase (XerD), a cell division protein, FtsK/SpoIIIE, and a fucose utilization operon first characterized in serotype 3 (SP3-BS71) (Higgins *et al.*, 2009). Unique to all of the ST618 isolates was a type 2 fucose utilization operon, first characterized in *S. pneumoniae* TIGR4 (Chan *et al.*, 2003) (Fig. S8). Fucose utilization operons have been shown to be essential to pneumococcal virulence in models of pneumonia and otitis media (Embry *et al.*, 2007). It is possible that the SP3-BS71-type operon found in ST3081 allows ST3081 to outcompete ST618 isolates, which possess the type 2 operon. Of the remaining proteins unique to ST3081, XerD has previously been implicated in pneumococcal virulence (Chalker *et al.*, 2000). FtsK has not previously been implicated in pneumococcal virulence; however, it interacts with Xer proteins (Le Bourgeois *et al.*, 2007), suggesting that FtsK may play an indirect role in the regulation of pneumococcal virulence.

## Discussion

The global serotype 1 population shows a strong phylogeographic structure at a continental level with further country-specific phylogeographic structure evident within Africa. The data suggest that serotype 1 has disseminated within Africa and diversified independently within the continent, likely due to country- and population-specific selective pressures. There is limited evidence of inter-country transfer events within the African continent, arguably due to the dominance of locally circulating clones, limiting the scope of new clones to become established within the population, and the short carriage duration of serotype 1 limiting opportunity for inter-country transfer. Yet, the relatively high rec/m reported here suggests that this serotype is carried for periods long enough to allow extensive recombination. We report levels of recombination in serotype 1 in line with other serotypes that are commonly associated with carriage and antimicrobial resistance (Chewapreecha *et al.*, 2014; Croucher *et al.*, 2011). Our data suggest that recombination and antimicrobial consumption have contributed to the diversification of serotype 1 in Africa. We observed recombination events in the targets of co-trimoxazole or SP and to a lesser extent fluoroquinolone and beta-lactam antimicrobials. The varying rates of recombination and frequency of recombination encompassing these genes between lineage B African clades show evidence of adaptation to different environmental pressures within geographically isolated clades. The distribution of transposable resistance elements in the accessory genome conferring tetracycline and chloramphenicol resistance further supports that antimicrobial usage has shaped diversification. The accessory genome of serotype 1 is highly variable between regions; region/clade-specific genes are implicated in virulence and evasion of the host immune response, suggesting that difference in host genetics between regions is also driving diversification. Furthermore, the phylogeny shows that long-range transmission of serotype 1 is rare. This lack of long-range transmission has led to the divergence of geographically isolated clades, which have remained stable with little impact from imported clones. In summary, our data show that serotype 1 is genetically distinct in Africa relative to other continents and furthermore is distinct between regions of Africa. Clonal replacement of the dominant serotype 1 clones circulating within regions is rare; however, we report the accessory gene content that has likely driven decisive serotype clonal replacement in the Gambia.

## Supplementary Data

3031 Supplementary Data

## References

[r1] Antonio M. Hakeem I. Awine T. Secka O. Sankareh K. Nsekpong D. Lahai G. Akisanya A. Egere U. other authors (2008). Seasonality and outbreak of a predominant *Streptococcus pneumoniae* serotype 1 clone from The Gambia: expansion of ST217 hypervirulent clonal complex in West Africa BMC Microbiol 8 198 10.1186/1471-2180-8-198.. 19014613 PMC2587476

[r2] Bentley S.D. Aanensen D.M. Mavroidi A. Saunders D. Rabbinowitsch E. Collins M. Donohoe K. Harris D. Murphy L. other authors (2006). Genetic analysis of the capsular biosynthetic locus from all 90 pneumococcal serotypes PLoS Genet 2 e31 10.1371/journal.pgen.0020031. 16532061 PMC1391919

[r3] Blumental S. Moïsi J.C. Roalfe L. Zancolli M. Johnson M. Burbidge P. Borrow R. Yaro S. Mueller J.E. other authors (2015). *Streptococcus**pneumoniae* serotype 1 burden in the African meningitis belt: exploration of functionality in specific antibodies Clin Vaccine Immunol 22 404–412 10.1128/CVI.00758-14. 25651921 PMC4375351

[r4] Boetzer M. Henkel C.V. Jansen H.J. Butler D. Pirovano W. (2011). Scaffolding pre-assembled contigs using sspace Bioinformatics 27 578–579 10.1093/bioinformatics/btq683. 21149342

[r5] Brueggemann A.B. Spratt B.G. (2003). Geographic distribution and clonal diversity of *Streptococcus pneumoniae* serotype 1 isolates J Clin Microbiol 41 4966–4970 10.1128/JCM.41.11.4966-4970.2003.. 14605125 PMC262517

[r6] Brueggemann A.B. Griffiths D.T. Meats E. Peto T. Crook D.W. Spratt B.G. (2003). Clonal relationships between invasive and carriage *Streptococcus pneumoniae* and serotype- and clone-specific differences in invasive disease potential J Infect Dis 187 1424–1432 10.1086/374624. 12717624

[r7] Chalker A.F. Lupas A. Ingraham K. So C.Y. Lunsford R.D. Li T. Bryant A. Holmes D.J. Marra A. other authors (2000). Genetic characterization of gram-positive homologs of the XerCD site-specific recombinases J Mol Microbiol Biotechnol 2 225–233 10939248

[r8] Chan P.F. O'Dwyer K.M. Palmer L.M. Ambrad J.D. Ingraham K.A. So C. Lonetto M.A. Biswas S. Rosenberg M. other authors (2003). Characterization of a novel fucose-regulated promoter (PfcsK) suitable for gene essentiality and antibacterial mode-of-action studies in *Streptococcus pneumoniae* J Bacteriol 185 2051–2058 10.1128/JB.185.6.2051-2058.2003. 12618474 PMC150135

[r9] Chewapreecha C. Harris S.R. Croucher N.J. Turner C. Marttinen P. Cheng L. Pessia A. Aanensen D.M. Mather A.E. other authors (2014). Dense genomic sampling identifies highways of pneumococcal recombination Nat Genet 46 305–309 10.1038/ng.2895. 24509479 PMC3970364

[r10] Cornick J.E. Bentley S.D. (2012). *Streptococcus pneumoniae*: the evolution of antimicrobial resistance to beta-lactams, fluoroquinolones and macrolides Microbes Infect 14 573–583 10.1016/j.micinf.2012.01.012. 22342898

[r11] Cornick J.E. Harris S.R. Parry C.M. Moore M.J. Jassi C. Kamng'ona A. Kulohoma B. Heyderman R.S. Bentley S.D. Everett D.B. (2014). Genomic identification of a novel co-trimoxazole resistance genotype and its prevalence amongst *Streptococcus pneumoniae* in Malawi J Antimicrob Chemother 69 368–374. 24080503 10.1093/jac/dkt384 PMC3886935

[r12] Croucher N.J. Harris S.R. Fraser C. Quail M.A. Burton J. van der Linden M. McGee L. von Gottberg A. Song J.H. other authors (2011). Rapid pneumococcal evolution in response to clinical interventions Science 331 430–434 10.1126/science.1198545. 21273480 PMC3648787

[r13] Croucher N.J. Finkelstein J.A. Pelton S.I. Mitchell P.K. Lee G.M. Parkhill J. Bentley S.D. Hanage W.P. Lipsitch M. (2013). Population genomics of post-vaccine changes in pneumococcal epidemiology Nat Genet 45 656–663 10.1038/ng.2625. 23644493 PMC3725542

[r14] Croucher N.J. Page A.J. Connor T.R. Delaney A.J. Keane J.A. Bentley S.D. Parkhill J. Harris S.R. (2015). Rapid phylogenetic analysis of large samples of recombinant bacterial whole genome sequences using Gubbins Nucleic Acids Res Nucleic Acids Res 43 e15. 25414349 10.1093/nar/gku1196 PMC4330336

[r15] Cutts F.T. Zaman S.M.A. Enwere G. Jaffar S. Levine O.S. Okoko J.B. Oluwalana C. Vaughan A. Obaro S.K. other authors (2005). Efficacy of nine-valent pneumococcal conjugate vaccine against pneumonia and invasive pneumococcal disease in The Gambia: randomised, double-blind, placebo-controlled trial Lancet 365 1139–1146 10.1016/S0140-6736(05)71876-6. 15794968

[r16] Dagan R. Gradstein S. Belmaker I. Porat N. Siton Y. Weber G. Janco J. Yagupsky P. (2000). An outbreak of *Streptococcus pneumoniae* serotype 1 in a closed community in southern Israel Clin Infect Dis 30 319–321 10.1086/313645. 10671335

[r17] Delcher A.L. Harmon D. Kasif S. White O. Salzberg S.L. (1999). Improved microbial gene identification with glimmer Nucleic Acids Res 27 4636–4641 10.1093/nar/27.23.4636. 10556321 PMC148753

[r18] Drummond A.J. Suchard M.A. Xie D. Rambaut A. (2012). Bayesian phylogenetics with BEAUti and the beast 1.7 Mol Biol Evol 29 1969–1973 10.1093/molbev/mss075. 22367748 PMC3408070

[r19] Embry A. Hinojosa E. Orihuela C.J. (2007). Regions of Diversity 8, 9 and 13 contribute to *Streptococcus pneumoniae* virulence BMC Microbiol 7 80 10.1186/1471-2180-7-80. 17723151 PMC2045101

[r20] Everett D.B. Mukaka M. Denis B. Gordon S.B. Carrol E.D. van Oosterhout J.J. Molyneux E.M. Molyneux M. French N. Heyderman R.S. (2011). Ten years of surveillance for invasive *Streptococcus pneumoniae* during the era of antiretroviral scale-up and cotrimoxazole prophylaxis in Malawi PLoS One 6 e17765 10.1371/journal.pone.0017765. 21423577 PMC3058053

[r21] Harboe Z.B. Benfield T.L. Valentiner-Branth P. Hjuler T. Lambertsen L. Kaltoft M. Krogfelt K. Slotved H.C. Christensen J.J. Konradsen H.B. (2010). Temporal trends in invasive pneumococcal disease and pneumococcal serotypes over 7 decades Clin Infect Dis 50 329–337 10.1086/649872. 20047478

[r22] Harris S.R. Feil E.J. Holden M.T. Quail M.A. Nickerson E.K. Chantratita N. Gardete S. Tavares A. Day N. other authors (2010). Evolution of MRSA during hospital transmission and intercontinental spread Science 327 469–474 10.1126/science.1182395. 20093474 PMC2821690

[r23] Hausdorff W.P. Feikin D.R. Klugman K.P. (2005). Epidemiological differences among pneumococcal serotypes Lancet Infect Dis 5 83–93 10.1016/S1473-3099(05)70083-9. 15680778

[r24] Higgins M.A. Abbott D.W. Boulanger M.J. Boraston A.B. (2009). Blood group antigen recognition by a solute-binding protein from a serotype 3 strain of *Streptococcus pneumoniae* J Mol Biol 388 299–309 10.1016/j.jmb.2009.03.012. 19285508 PMC2911475

[r25] Inouye M. Conway T.C. Zobel J. Holt K.E. (2012). Short read sequence typing (SRST): multi-locus sequence types from short reads BMC Genomics 13 338 10.1186/1471-2164-13-338. 22827703 PMC3460743

[r26] Iyer J.K. Milhous W.K. Cortese J.F. Kublin J.G. Plowe C.V. (2001). *Plasmodium falciparum* cross-resistance between trimethoprim and pyrimethamine Lancet 358 1066–1067 10.1016/S0140-6736(01)06201-8. 11589941

[r27] Johnson H.L. Deloria-Knoll M. Levine O.S. Stoszek S.K. Freimanis Hance L. Reithinger R. Muenz L.R. O'Brien K.L. (2010). Systematic evaluation of serotypes causing invasive pneumococcal disease among children under five: the pneumococcal global serotype project PLoS Med 7 e1000348 10.1371/journal.pmed.1000348. 20957191 PMC2950132

[r28] Klugman K.P. Madhi S.A. Huebner R.E. Kohberger R. Mbelle N. Pierce N. Vaccine Trialists Group (2003). A trial of a 9-valent pneumococcal conjugate vaccine in children with and those without HIV infection N Engl J Med 349 1341–1348 10.1056/NEJMoa035060. 14523142

[r29] Klugman K. Cutts F. Adegbola R.A. Black S. Madhi S.A. O'Brien K. Santosham M. Shinefield H. (2008). Meta-analysis of the efficacy of conjugate vaccines against invasive pneumococcal disease. In Pneumococcal Vaccines, pp. 317–326. Edited by Siber G. Klugman K. Mäkelä P. Washington, DC American Society for Microbiology 10.1128/9781555815820.ch21.

[r30] Köser C.U. Holden M.T. Ellington M.J. Cartwright E.J. Brown N.M. Ogilvy-Stuart A.L. Hsu L.Y. Chewapreecha C. Croucher N.J. other authors (2012). Rapid whole-genome sequencing for investigation of a neonatal MRSA outbreak N Engl J Med 366 2267–2275 10.1056/NEJMoa1109910. 22693998 PMC3715836

[r31] Le Bourgeois P. Bugarel M. Campo N. Daveran-Mingot M.L. Labonté J. Lanfranchi D. Lautier T. Pagès C. Ritzenthaler P. (2007). The unconventional Xer recombination machinery of streptococci/lactococci PLoS Genet 3 e117. 17630835 10.1371/journal.pgen.0030117 PMC1914069

[r32] Letunic I. Bork P. (2011). Interactive Tree Of Life v2: online annotation and display of phylogenetic trees made easy Nucleic Acids Res 39 W475–W478 10.1093/nar/gkr201. 21470960 PMC3125724

[r33] Li H. Durbin R. (2009). Fast and accurate short read alignment with Burrows-Wheeler transform Bioinformatics 25 1754–1760 10.1093/bioinformatics/btp324. 19451168 PMC2705234

[r34] Li L. Stoeckert C.J. Jr Roos D.S. (2003). OrthoMCL: identification of ortholog groups for eukaryotic genomes Genome Res 13 2178–2189 10.1101/gr.1224503. 12952885 PMC403725

[r35] Li M.-F. Zhang B.-C. Li J. Sun L. (2014). Sil: a *Streptococcus iniae* bacteriocin with dual role as an antimicrobial and an immunomodulator that inhibits innate immune response and promotes *S. iniae* infection PLoS One 9 e96222 10.1371/journal.pone.0096222. 24781647 PMC4004548

[r36] Mukouhara T. Arimoto T. Cho K. Yamamoto M. Igarashi T. (2011). Surface lipoprotein PpiA of Streptococcus mutans suppresses scavenger receptor MARCO-dependent phagocytosis by macrophages Infect Immun 79 4933–4940 10.1128/IAI.05693-11. 21986627 PMC3232644

[r37] O'Brien K.L. Wolfson L.J. Watt J.P. Henkle E. Deloria-Knoll M. McCall N. Lee E. Mulholland K. Levine O.S. Cherian T. Hib and Pneumococcal Global Burden of Disease Study Team (2009). Burden of disease caused by *Streptococcus pneumoniae* in children younger than 5 years: global estimates Lancet 374 893–902 10.1016/S0140-6736(09)61204-6. 19748398

[r38] Pai R. Moore M.R. Pilishvili T. Gertz R.E. Whitney C.G. Beall B. Active Bacterial Core Surveillance Team (2005). Postvaccine genetic structure of *Streptococcus pneumoniae* serotype 19A from children in the United States J Infect Dis 192 1988–1995 10.1086/498043. 16267772

[r39] United Nations High Commissioner for Refugees (2000). The State of the World's Refugees, 2000: Fifty Years of Humanitarian Action Oxford Oxford University Press.

[r40] Ritchie N.D. Mitchell T.J. Evans T.J. (2012). What is different about serotype 1 pneumococci? Future Microbiol 7 33–46 10.2217/fmb.11.146. 22191445

[r41] Seemann T. (2014). Prokka: rapid prokaryotic genome annotation Bioinformatics 30 2068–2069 10.1093/bioinformatics/btu153. 24642063

[r42] Stamatakis A. (2006). RAxML-VI-HPC: maximum likelihood-based phylogenetic analyses with thousands of taxa and mixed models Bioinformatics 22 2688–2690 10.1093/bioinformatics/btl446. 16928733

[r43] Turner P. Turner C. Jankhot A. Helen N. Lee S.J. Day N.P. White N.J. Nosten F. Goldblatt D. (2012). A longitudinal study of *Streptococcus pneumoniae* carriage in a cohort of infants and their mothers on the Thailand-Myanmar border PLoS One 7 e38271 10.1371/journal.pone.0038271. 22693610 PMC3365031

[r44] Williams T.M. Loman N.J. Ebruke C. Musher D.M. Adegbola R.A. Pallen M.J. Weinstock G.M. Antonio M. (2012). Genome analysis of a highly virulent serotype 1 strain of *Streptococcus pneumoniae* from West Africa PLoS One 7 e26742 10.1371/journal.pone.0026742. 23082106 PMC3474768

[r45] Yildirim I. Hanage W.P. Lipsitch M. Shea K.M. Stevenson A. Finkelstein J. Huang S.S. Lee G.M. Kleinman K. Pelton S.I. (2010). Serotype specific invasive capacity and persistent reduction in invasive pneumococcal disease Vaccine 29 283–288 10.1016/j.vaccine.2010.10.032. 21029807 PMC3139683

[r46] Zerbino D.R. Birney E. (2008). Velvet: algorithms for de novo short read assembly using de Bruijn graphs Genome Res 18 821–829 10.1101/gr.074492.107. 18349386 PMC2336801

