# Supplementary material for: Region-specific diversification of the highly virulent serotype 1 Streptococcus pneumoniae
Source: Microb Genom. 2015 Aug 11;1(2):e000027. doi: 10.1099/mgen.0.000027 (PMC5320570; doi:10.1099/mgen.0.000027)
Supplement: Supplementary file 1 — Supplementary Data [file mgen-01-27-s001.docx]

**Supplementary material**

**Sampling Strategy**

We sequenced a combined study collection of 448 isolates. The collection included 179 serotype 1 pneumococci recovered from hospital, surveillance and carriage studies across four main African study sites (The Gambia n=50, Malawi n=71, Niger n=39 and South Africa n=58):

**The Gambia**

Invasive isolates were collected from two regions in the Gambia:

1. The Western region (MRC clinic) – all patients presenting with symptoms suggestive pneumonia or meningitis are routinely given a blood culture. Seventeen (17) of the 58 (29%) serotype 1 pneumococci isolated from BC (2003-2008), were included in the study collection.
2. The Upper River region (Basse Health Centre) – isolates were collected from patients with suspected, pneumonia, meningitis and sepsis as part of a population-based surveillance study into PCV effectiveness. The study started in 2007, 38 serotype 1 pneumococci were available 2007-2008, of which 15 (39%) were included in the study collection.

Carriage isolates were collected in the Western region as part of two large pneumococcal carriage studies (2003-2009) [[1](#_ENREF_1),[2](#_ENREF_2)]. Eight seven (87) serotype 1 isolates were available, 18 (21%) were included in the study collection.

**Malawi**

Isolates were obtained via the routine hospital diagnostics at Queen Elizabeth Central Hospital (QECH), a 1250 government-funded hospital serving a population of ~1 million, including Blantyre, the surrounding township and villages. All general medicine admission presenting with evidence of sepsis, have blood drawn for culture. Blood is also drawn for culture from all febrile children admitted to QECH that have a negative thick film for parasites, children regarded to be severely ill with meningitis or sepsis regardless of their malaria film, or children that did not respond to initial malaria treatment and remained febrile. CSF is collected from all adult general admissions with clinically suspected meningitis or children that received a BC. Pneumococci isolated from blood or CSF are stored in the Malawi-Liverpool-Wellcome Trust Strain archive, the archive houses >5000 pneumococcal isolates collected from 1997 to date. One hundred and fifty (150) isolates were randomly selected each year from 2003-2008 and screened for serotype 1 by serotype 1 specific colony PCR. 180 of the screened isolates were serotype 1. Seventy-one (39%) of these isolates remained viable for genomic DNA extraction and were included in the study collection.

**Niger**

Invasive isolates were obtained from the National Reference Laboratory (NRL) for meningitis. The MoPH of the Republic of Niger reported a total of 23,818 suspected cases of meningitis 2003-2008; CSF was obtained from 11,883 (49.9%) of these cases. Among those CSF, 763 were *lytA* positive, however 56 subsequently went missing or the CSF volume was too low for further characterisation. The remaining 707, *lytA* positive samples were screened with a simplex PCR for *cpsA* genes. A total of 53 CSF were negative for *cpsA*. The remaining 654 were subjected serotyping by sequential multiplex PCR; 297 (45.4%) were identified as serotype 1. Due to CSF arriving either unrefrigerated or unfrozen, only 39 of the serotype 1 pneumococci could be cultured. These 39 were all included in the study collection.

**South Africa**

Active, laboratory-based national surveillance of invasive pneumococcal disease (IPD) is conducted through the Group for Enteric, Respiratory and Meningitis surveillance in South Africa (GERMS-SA). Over 130 laboratories report cases of IPD to this system, representing ~290 hospitals throughout South Africa. Normally sterile specimens yielding pneumococci are reported and isolates are serotyped by Quellung reaction using specific anti-sera. From 2004-2008, GERMS-SA recorded 2705 cases of serotype 1 IPD. A random selection of 58 of these was included in the study collection.

To provide phylogeographic context, a convenience sample of 234 isolates provided by the CDC Global Pneumococcal Strain Bank Project (n=199) and Swiss Tropical Institute (n=35) were also sequenced which included isolates from an additional seven African countries (n=145) and 16 non-African countries, spanning four other continents (n=124) (Supplementary Table S1, Supplementary Figure S1). All isolates were collected between 1994 and 2009, prior to the widespread roll out of the PCV in Africa.

The isolates were recovered from cases of disease (blood, 54%; CSF, 16%; pleural fluid, 1%; sputum, 1%; throat swab, 1%) and nasopharyngeal carriage in healthy individuals (6%). Information on the origin of 5% of the study isolates (n=22) could not be obtained. The isolates were recovered from individuals ranging from 1 month to 83 years of age. The percentage of isolates in each of the four age groups was: ≤2 years, 12%; ≤5 years, 6%; <15 years, 15%; ≥15 years, 23%. Age was unavailable for 43% (n=193) of the isolates. The isolates were collected to encompass diversity with respect to geographic location, date of isolation, clinical source and patient age (Supplementary Table S1).

**Table S1**. Details of *S. pneumoniae* serotype 1 isolates included in the study

|  |  |  | **No. of isolates of the following specimen type** | | | | | | | **No. of isolates in the following age group** | | | | | **No. of isolates which were collected in the following context** | | | |
| --- | --- | --- | --- | --- | --- | --- | --- | --- | --- | --- | --- | --- | --- | --- | --- | --- | --- | --- |
| **Continent** | **Country** | **No. of isolates** | **Blood** | **CSF** | **Pleural Fluid** | **Sputum** | **Throat Swab** | **NP Carriage** | **Unknown** | **≤2** | **≤5** | **5 to 15** | **15>** | **Unknown** | **Hospital** | **Surveillance** | **Carriage studies** | **Unknown** |
| **Africa** | **Egypt** | 7 |  | 7 |  |  |  |  |  |  |  |  |  | 7 |  |  |  | 7 |
|  | **Ethiopia** | 2 |  |  |  |  |  | 2 |  |  |  |  |  | 2 |  |  |  | 2 |
|  | **Ghana** | 35 |  | 35[35] |  |  |  |  |  | 3 | 3 | 8 | 18 | 3 |  |  |  | 35 |
|  | **Malawi** | 71 | 38 | 32 |  |  |  | 1 |  | 11 | 7 | 16 | 31 | 6 | 71 |  |  |  |
|  | **Mozambique** | 50 | 47 | 3 |  |  |  |  |  |  |  |  |  | 50 |  |  |  | 50 |
|  | **Niger** | 39 |  | 39[5] |  |  |  |  |  | 7 | 2 | 9 | 21 |  |  | 39 |  |  |
|  | **Nigeria** | 2 |  |  |  |  |  | 2 |  |  | 1 | 1 |  |  |  |  | 2 |  |
|  | **Senegal** | 4 |  | 4 |  |  |  |  |  | 2 | 1 |  | 1 |  | 4 |  |  |  |
|  | **South Africa** | 58 | 43 | 14 |  |  |  |  | 1 | 13 | 8 | 18 | 19 |  |  | 58 |  |  |
|  | **The Gambia** | 50 | 29 | 1 | 2 |  |  | 18 |  | 12 | 7 | 15 | 15 | 1 | 17 | 15 | 18 |  |
|  | **Togo** | 6 |  | 6 |  |  |  |  |  | 6 |  |  |  |  |  | 6 |  |  |
|  |  |  |  |  |  |  |  |  |  |  |  |  |  |  |  |  |  |  |
| **Europe** | **Croatia** | 2 | 2 |  |  |  |  |  |  |  |  |  |  | 2 |  |  |  | 2 |
|  | **Slovenia** | 1 | 1 |  |  |  |  |  |  |  |  |  |  | 1 |  |  |  | 1 |
|  | **Spain** | 8 | 8 |  |  |  |  |  |  |  |  |  |  | 8 |  |  |  | 8 |
|  | **Sweden** | 17 | 17 |  |  |  |  |  |  |  |  |  |  | 17 |  |  |  | 17 |
|  |  |  |  |  |  |  |  |  |  |  |  |  |  |  |  |  |  |  |
| **Asia** | **Bangladesh** | 7 | 7 |  |  |  |  |  |  |  |  |  |  | 7 |  |  |  | 7 |
|  | **China** | 8 |  |  |  | 2 |  |  | 6 |  |  |  |  | 8 |  |  |  | 8 |
|  | **Hong Kong** | 1 | 1 |  |  |  |  |  |  |  |  |  |  | 1 |  |  |  | 1 |
|  | **India** | 17 | 1 | 1 |  |  |  |  | 15 |  |  |  |  | 17 |  |  |  | 17 |
|  | **Nepal** | 4 |  |  |  |  |  | 4 |  |  |  |  |  | 4 |  |  |  | 4 |
|  | **Philippines** | 3 | 1 | 1 |  |  |  |  | 1 |  |  |  |  | 3 |  |  |  | 3 |
|  | **Qatar** | 9 | 9 |  |  |  |  |  |  |  |  |  |  | 9 |  |  |  | 9 |
|  | **Thailand** | 27 | 20 | 1 |  | 2 | 4 |  |  |  |  |  |  | 27 |  |  |  | 27 |
|  |  |  |  |  |  |  |  |  |  |  |  |  |  |  |  |  |  |  |
| **South America** | **Argentina** | 6 | 6 |  |  |  |  |  |  |  |  |  |  | 6 |  |  |  | 6 |
|  | **Brazil** | 9 | 7 |  | 2 |  |  |  |  |  |  |  |  | 9 |  |  |  | 9 |
|  | **Peru** | 2 | 1 |  | 1 |  |  |  |  |  |  |  |  | 2 |  |  |  | 2 |
|  |  |  |  |  |  |  |  |  |  |  |  |  |  |  |  |  |  |  |
| **New Zealandia** | **New Zealand** | 3 | 3 |  |  |  |  |  |  |  |  |  |  | 3 |  |  |  | 3 |
|  |  |  |  |  |  |  |  |  |  |  |  |  |  |  |  |  |  |  |
|  | **Total** | 448 | 241 | 70 | 5 | 4 | 4 | 27 | 23 | 54 | 29 | 67 | 105 | 193 | 92 | 118 | 20 | 218 |
|  | **Percentage** | 100 | 54 | 16 | 1 | 1 | 1 | 6 | 5 | 12 | 6 | 15 | 23 | 43 | 21 | 26 | 4 | 49 |

**Table S2**. MLST profiles of the *S. pneumoniae* serotype 1 isolates included in the study

|  |  | **No. of isolates of the following ST** | | | | | | | | | | | | | | | | | | | | | | | | | | | |  |  |
| --- | --- | --- | --- | --- | --- | --- | --- | --- | --- | --- | --- | --- | --- | --- | --- | --- | --- | --- | --- | --- | --- | --- | --- | --- | --- | --- | --- | --- | --- | --- | --- |
| **Continent of origin** | **Country of origin** | **DLV of 217** | **ST2034** | **ST618** | **ST217*** | **Novel** | **ST303** | **ST2084** | **ST4755** | **ST8158** | **SLV of 217** | **ST5632** | **ST7712** | **ST2206** | **ST2839** | **ST3081** | **ST612** | **ST228** | **ST306*** | **ST304*** | **SLV of 304** | **ST2296** | **SLV of ST2296** | **ST3044** | **ST5002** | **ST5044** | **ST5672** | **ST3018** | **ST615** | **Total no. of isolates** | **Percentage** |
| **Africa** | **Egypt** | 3 | 1 | 3 |  |  |  |  |  |  |  |  |  |  |  |  |  |  |  |  |  |  |  |  |  |  |  |  |  | 7 | 1.6 |
|  | **Ethiopia** |  |  |  | 2 |  |  |  |  |  |  |  |  |  |  |  |  |  |  |  |  |  |  |  |  |  |  |  |  | 2 | 0.4 |
|  | **Ghana** |  |  |  | 3 | 1 | 27 | 2 | 2 |  |  |  |  |  |  |  |  |  |  |  |  |  |  |  |  |  |  |  |  | 35 | 7.8 |
|  | **Malawi** |  |  |  | 60 |  | 2 |  |  | 1 | 8 |  |  |  |  |  |  |  |  |  |  |  |  |  |  |  |  |  |  | 71 | 15.8 |
|  | **Mozambique** |  |  |  | 41 | 4 |  |  |  |  | 3 | 1 | 1 |  |  |  |  |  |  |  |  |  |  |  |  |  |  |  |  | 50 | 11.2 |
|  | **Niger** |  |  |  | 21 |  | 15 |  |  |  |  |  |  | 2 | 1 |  |  |  |  |  |  |  |  |  |  |  |  |  |  | 39 | 8.7 |
|  | **Nigeria** |  |  |  | 2 |  |  |  |  |  |  |  |  |  |  |  |  |  |  |  |  |  |  |  |  |  |  |  |  | 2 | 0.4 |
|  | **Senegal** |  |  |  |  |  |  |  |  |  |  |  |  |  |  | 4 |  |  |  |  |  |  |  |  |  |  |  |  |  | 4 | 0.9 |
|  | **South Africa** |  |  |  | 49 |  |  |  |  |  |  |  |  |  |  |  | 9 |  |  |  |  |  |  |  |  |  |  |  |  | 58 | 12.9 |
|  | **The Gambia** |  |  | 6 | 4 | 1 | 2 | 1 |  |  |  |  |  |  |  | 36 |  |  |  |  |  |  |  |  |  |  |  |  |  | 50 | 11.2 |
|  | **Togo** |  |  |  |  |  | 6 |  |  |  |  |  |  |  |  |  |  |  |  |  |  |  |  |  |  |  |  |  |  | 6 | 1.3 |
|  |  |  |  |  |  |  |  |  |  |  |  |  |  |  |  |  |  |  |  |  |  |  |  |  |  |  |  |  |  |  | 0.0 |
| **Europe** | **Croatia** |  |  |  |  |  |  |  |  |  |  |  |  |  |  |  |  | 1 | 1 |  |  |  |  |  |  |  |  |  |  | 2 | 0.4 |
|  | **Slovenia** |  |  |  |  |  |  |  |  |  |  |  |  |  |  |  |  |  |  | 1 |  |  |  |  |  |  |  |  |  | 1 | 0.2 |
|  | **Spain** |  |  |  |  |  |  |  |  |  |  |  |  |  |  |  |  | 3 | 1 | 4 |  |  |  |  |  |  |  |  |  | 8 | 1.8 |
|  | **Sweden** |  |  |  |  | 2 |  |  |  |  |  |  |  |  |  |  |  |  | 14 |  | 1 |  |  |  |  |  |  |  |  | 17 | 3.8 |
|  |  |  |  |  |  |  |  |  |  |  |  |  |  |  |  |  |  |  |  |  |  |  |  |  |  |  |  |  |  |  | 0.0 |
| **Asia** | **Bangladesh** |  |  |  |  |  | 7 |  |  |  |  |  |  |  |  |  |  |  |  |  |  |  |  |  |  |  |  |  |  | 7 | 1.6 |
|  | **China** |  |  |  |  |  |  |  |  |  |  |  |  |  |  |  |  |  |  |  |  | 7 | 1 |  |  |  |  |  |  | 8 | 1.8 |
|  | **Hong Kong** |  |  |  |  | 1 |  |  |  |  |  |  |  |  |  |  |  |  |  |  |  |  |  |  |  |  |  |  |  | 1 | 0.2 |
|  | **India** |  |  |  | 4 | 2 |  |  |  |  |  |  |  |  | 1 |  |  |  |  |  |  |  |  | 3 | 1 | 3 | 3 |  |  | 17 | 3.8 |
|  | **Nepal** |  |  |  |  |  | 4 |  |  |  |  |  |  |  |  |  |  |  |  |  |  |  |  |  |  |  |  |  |  | 4 | 0.9 |
|  | **Philippines** | 1 |  |  | 1 |  |  |  |  |  |  |  |  |  |  | 1 |  |  |  |  |  |  |  |  |  |  |  |  |  | 3 | 0.7 |
|  | **Qatar** |  |  |  | 1 | 1 | 4 |  |  |  |  |  |  |  |  |  |  |  | 1 |  |  |  |  |  |  |  |  | 2 |  | 9 | 2.0 |
|  | **Thailand** | 2 |  |  | 13 |  | 4 |  |  |  |  |  |  |  |  |  |  |  |  |  |  |  |  |  |  |  |  | 8 |  | 27 | 6.0 |
|  |  |  |  |  |  |  |  |  |  |  |  |  |  |  |  |  |  |  |  |  |  |  |  |  |  |  |  |  |  |  | 0.0 |
| **South America** | **Argentina** |  |  |  |  | 1 |  |  |  |  |  |  |  |  |  |  |  |  |  |  |  |  |  |  |  |  |  |  | 5 | 6 | 1.3 |
|  | **Brazil** |  |  |  |  |  |  |  |  |  |  |  |  |  |  |  |  |  |  | 8 | 1 |  |  |  |  |  |  |  |  | 9 | 2.0 |
|  | **Peru** |  |  |  |  | 1 |  |  |  |  |  |  |  |  |  |  |  |  |  |  |  |  |  |  |  |  |  |  | 1 | 2 | 0.4 |
|  |  |  |  |  |  |  |  |  |  |  |  |  |  |  |  |  |  |  |  |  |  |  |  |  |  |  |  |  |  |  | 0.0 |
| **New Zealandia** | **New Zealand** |  |  |  |  |  |  |  |  |  |  |  |  |  |  |  |  |  |  | 3 |  |  |  |  |  |  |  |  |  | 3 | 0.7 |
|  | **Total no. of isolates** | 6 | 1 | 9 | 201 | 14 | 71 | 3 | 2 | 1 | 11 | 1 | 1 | 2 | 2 | 41 | 9 | 4 | 17 | 16 | 2 | 7 | 1 | 3 | 1 | 3 | 3 | 10 | 6 | 448 | 100.0 |
|  | **Percentage** | 1.3 | 0.2 | 2.0 | 44.9 | 3.1 | 15.8 | 0.7 | 0.4 | 0.2 | 2.5 | 0.2 | 0.2 | 0.4 | 0.4 | 9.2 | 2.0 | 0.9 | 3.8 | 3.6 | 0.4 | 1.6 | 0.2 | 0.7 | 0.2 | 0.7 | 0.7 | 2.2 | 1.3 | 100.0 |  |

**Table S3.** Genes subject to recent recombination events (see separate excel file)

**Table S4.** Minimum inhibitory concentration (MIC) data to a panel of 15 antimicrobials was available for a subset of the study isolates (n=210).

|  | **Minimum inhibitory concentration**  **(mg/mL)** | | | | | **No of isolates (%)** | | | **Breakpoint values**  **(mg/mL)** | | |
| --- | --- | --- | --- | --- | --- | --- | --- | --- | --- | --- | --- |
| **Antimicrobial** | **Min** | **Max** | **MIC_50_** | **MIC_90_** | **GM** | **S** | **I** | **R** | **S** | **I** | **R** |
| **Benzyl Penicillin** | 0.03 | 0.06 | 0.03 | 0.03 | 0.03 |  |  |  |  |  |  |
| **Meningitis breakpoints** |  |  |  |  |  | 210 (100) | - | 0 (0) | 0.06≤ | - | ≥0.12 |
| **Non-meningitis breakpoints** |  |  |  |  |  | 210 (100) | 0 (0) | 0 (0) | 2≤ | 4 | ≥8 |
| **Ceftriaxone** | 0.05 | 1 | 0.5 | 0.5 | 0.5 |  |  |  |  |  |  |
| **Meningitis breakpoints** |  |  |  |  |  | 210 (99) | 1 (1) | 0 (0) | 0.5≤ | 1 | ≥2 |
| **Non-meningitis breakpoints** |  |  |  |  |  | 210 (100) | 0 (0) | 0 (0) | 1≤ | 2 | ≥4 |
| **Amoxicillin** | 0.03 | 0.06 | 0.03 | 0.03 | 0.03 |  |  |  |  |  |  |
| **Meningitis breakpoints** |  |  |  |  |  | 210 (1) | 0 (0) | 0 (0) | 2≤ | 4 | ≥8 |
| **Vancomycin** | 0.25 | 1 | 0.5 | 0.5 | 0.5 | 210 (11) | - | - | 1≤ | - | - |
| **Chloramphenicol** | 2 | 8 | 4 | 8 | 4 | 140 (67) | - | 70 (33) | 4≤ | - | ≥8 |
| **Ciprofloxacin*** | 2 | 4 | 2 | 2 | 2 | 0 (0) | 197 (94) | 14 (6) | 0.12≤ | 0.25-2 | >2 |
| **Levofloxacin** | 0.5 | 2 | 1 | 2 | 1 | 210 (100) | 0 (0) | 0 (0) | 2≤ | 4 | ≥8 |
| **Erythromycin** | 0.03 | 0.06 | 0.06 | 0.06 | 0.05 | 210 (100) | 0 (0) | 0 (0) | 0.25≤ | 0.5 | ≥1 |
| **Cotrimoxazole** | 0.25 | 4 | 2 | 4 | 1.5 | 102 (48) | 16 (8) | 92 (44) | 1≤ | 2 | >2 |
| **Tetracycline** | 2 | 8 | 8 | 8 | 4 | 101 (48) | 0 (0) | 109 (52) | 2≤ | 4 | ≥8 |
| MIC50, 50% MIC; MIC90, 90% MIC; GM, geometric mean; S, susceptible; I, intermediate; R, resistant; –, no intermediate resistance values for these antimicrobials. *All breakpoint values are taken from 2012 CLSI guidelines with the exception of ciprofloxacin breakpoints, taken from the BSAC 2009 guidelines.  A single South African isolates exhibited intermediate resistance to ceftriaxone based on meningitis breakpoints (48/A300907)  All isolates exhibited uniform MICs to rifampicin (1 μg/mL), linezolid (2 μg/mL) and synercid (1 μg/mL) | | | | | | | | | | | |

**Table S5.** Percentage distribution of accessory genes between clades (see separate excel file)

**Table S6.** Assembly statistics and ERS accession numbers for the study isolates (see separate excel file)

**Figure S1.** Plot showing the distribution of mean sequencing depth of mapped Illumina reads to a reference genome. Isolates are ranked in sequencing depth descending order. The maximum mapping depth of coverage observed was 573x; the minimum mapping depth of coverage observed was 23x.

**Figure S2**. Map showing the geographic origin of the study isolates. Green shading represents the four main study sites: Malawi, The Gambia, South Africa and Niger. Pink represents countries within Africa that contributed isolates to the study. Orange represents countries outside of Africa that contributed isolates to the study.


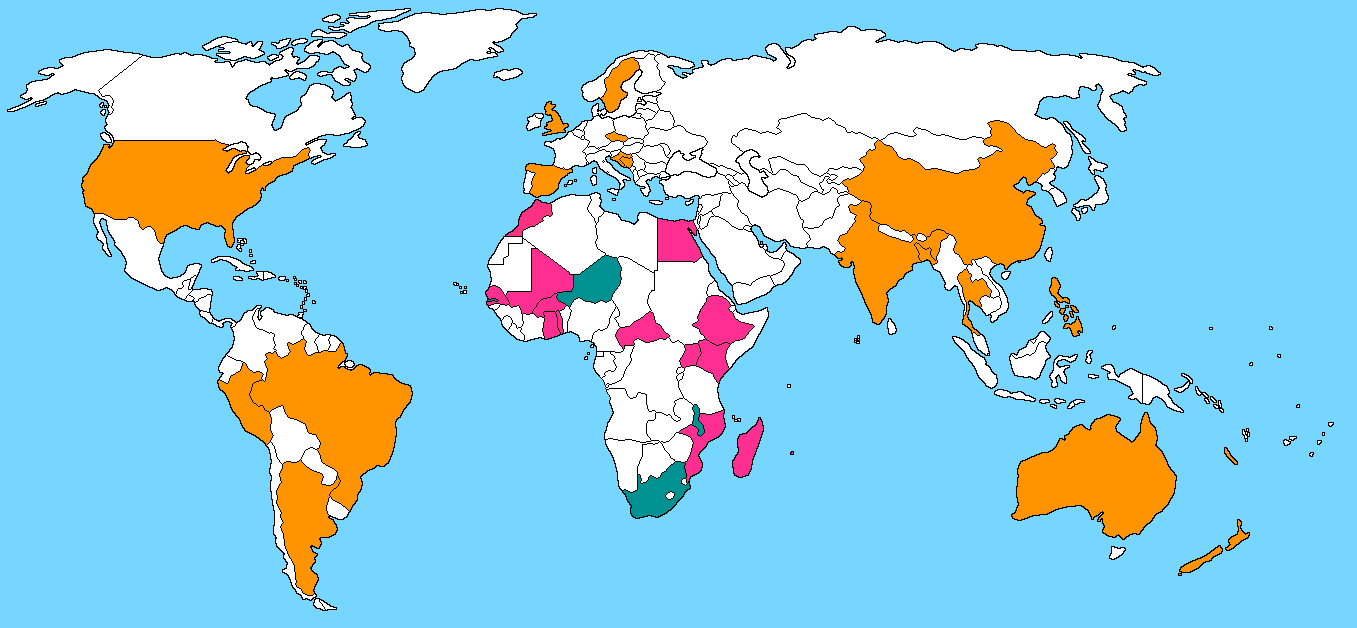


**Figure S3**. Map showing the African countries/regions represented in Lineage B.

**Figure S4. Terminal branch lengths of the five African clades within lineage B.**

The terminal branch length between each isolate and its last common ancestor was determined from the phylogeny illustrated in Figure 2.

| **Clade** | **Mean Terminal Branch Length** |
| --- | --- |
| 1 | 16.77273 |
| 2 | 13.06494 |
| 3 | 14.99242 |
| 4 | Not included in analysis |
| 5 | 3.860465 |
| 6 | 6.6667 |

**Figure S5**. Lineage B phylogeny annotated with the isolates Multilocus Sequence Type.

**Figure S6. Recombinant block sizes.**

The frequency of the different size recombinant blocks detected in the lineage B isolates.

**Figure S7. Core genome size decreasing**. The lineage B core genomes based on 376 pneumococcal isolates. A box and whisker plot of the number of core gene orthologous clusters observed as the subset of isolates included in the analysis increases, where the subset ranged from 1 to the total number of isolates (*n*=376). To generate a subset of isolates, *n* isolates are randomly selected from the dataset. The core genome size is then calculated for *n* isolates. Each random subset of a given size is generated 100 times.

**
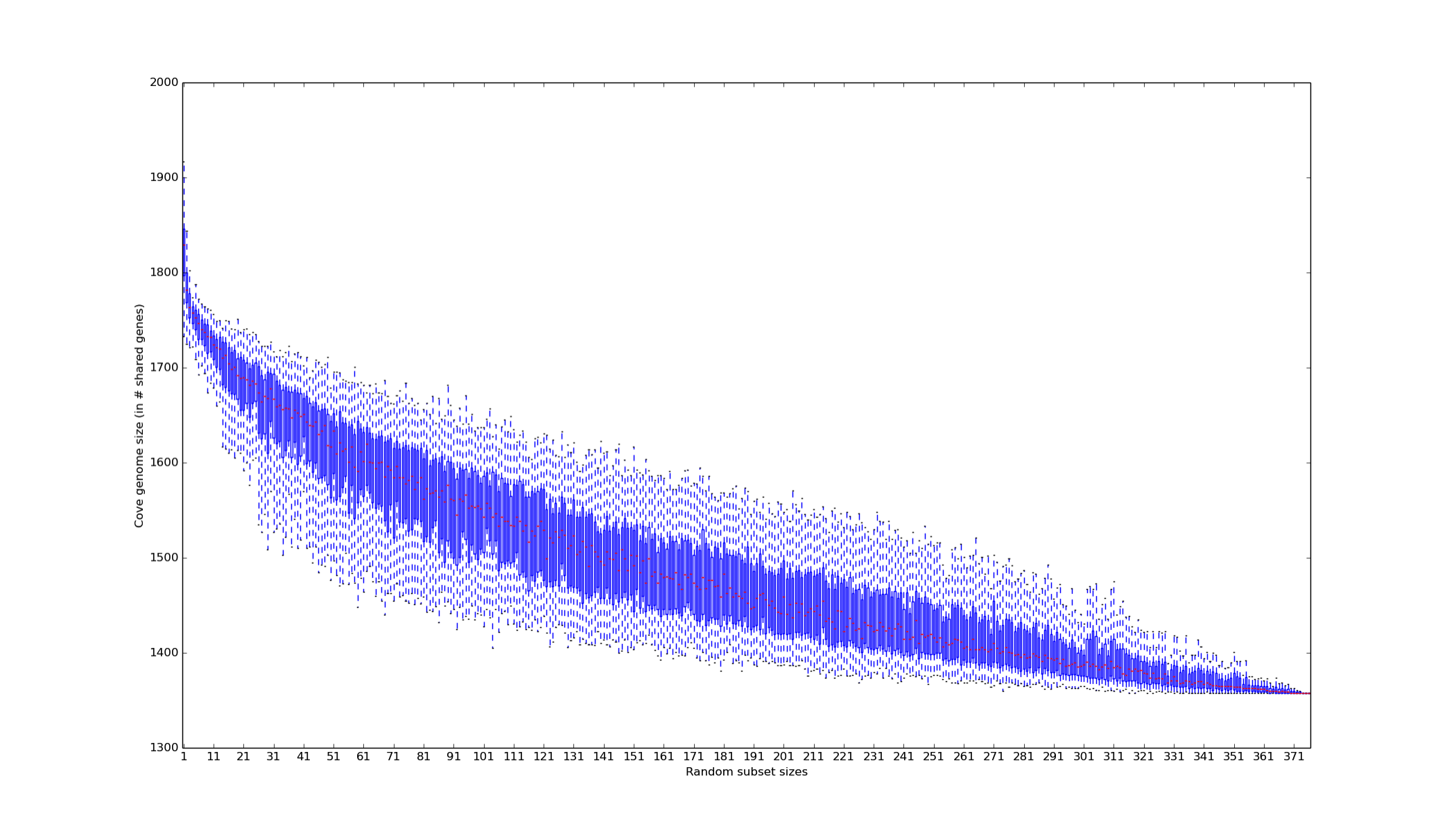
**

**Figure S8.** The two fucose utilization operons identified in *S. pneumoniae* isolated from the Gambia. (a) A type 2 fucose utilization operon that was first described in *S. pneumoniae* TIGR4 was identified in all of the ST618 isolates. (b) An alternative fucose utilization operon first described in SP3-BS71 was identified in all of the ST3081 study isolates. Grey blocks show regions of nucleotide homology between the two operons. Both operons are comprised of 11 genes; three of which show high nucleotide identity (FcsR, FcsK, Fscl).

The Pneumococcal African Genome project (PAGe) is a consortium funded by the Bill and Melinda Gates Foundation focused on genomic analysis of the pneumococcus across Africa. Further details can be found at http://www.pagegenomes.org/

### Partners

- Malawi-Liverpool-Wellcome Trust Clinical Research Programme (MLW), Malawi, [Link](http://www.mlw.medcol.mw/)
- Centre de Recherche Médicale et Sanitaire (CERMES), Niger, [Link](http://www.cermes.net/cermes/)
- National Institute for Communicable Diseases (NICD) of the National Health Laboratory Service (NHLS), South Africa, [Link](http://www.nicd.ac.za/)
- Medical Research Council (MRC), The Gambia, [Link](http://www.mrc.gm/)
- Wellcome Trust Sanger Institute (WTSI), UK, [Link](http://www.sanger.ac.uk/)
- University of Liverpool, UK, [Link](http://www.liv.ac.uk/)
- Liverpool School of Tropical Medicine, UK, [Link](http://www.lstmliverpool.ac.uk/)
- Wellcome Trust/LEPRA Karonga Prevention Programme (KPS), Malawi, [Link](http://www.lshtm.ac.uk/eph/ide/research/kps/)
- London School of Hygiene & Tropical Medicine, UK, [Link](http://www.lshtm.ac.uk/)
- Emory University, USA, [Link](http://www.pagegenomes.org/page/www.emory.edu/)

### New Partners

- Medical Research Council (MRC), Uganda, [Link](http://www.mrcuganda.org/)
- Centre for Disease Control (CDC), Kenya, [Link](http://www.pagegenomes.org/page/consortium)
- Agence de Medecine Preventive (AMP), Burkina Faso, [Link](http://www.amp-vaccinology.org/)
- Institut Pasteur du Maroc, [Link](http://www.pasteur.ma/)
- Institut Pasteur de Nouvelle-Calédonie, [Link](http://www.institutpasteur.nc/)
- Institut Pasteur de Madagascar, [Link](http://www.pasteur.mg/)
- Swiss Tropical and Public Health Institute (Swiss TPH), Switzerland, [Link](http://www.swisstph.ch/)
- CDC, Active Bacterial Core surveillance (ABCs), USA, [Link](http://www.cdc.gov/abcs/index.html)

### Collaborating Institutions

- Wellcome Trust Tropical Centre, Liverpool, UK, [Link](http://www.lstmliverpool.ac.uk/research/research-environment/wellcome-trust-tropical-centre)
- Institut Pasteur, Paris, France, [Link](http://www.pasteur.fr/ip/easysite/pasteur/en)

### Consortium Steering Group

- Dr Dean Everett, Malawi
- Dr Anne von Gottberg, South Africa
- Prof Keith Klugman, South Africa
- Dr Stephen D. Bentley, UK
- Dr Jean-Marc Collard, Niger
- Dr Martin Antonio, The Gambia
- Prof Rob Heyderman, Malawi
- Prof Neil French, UK

### Advisory Board

- Prof Sir Brian Greenwood (Chair), LSHTM, UK
- Dr Bill Hanage, Harvard, USA
- Prof Orin Levin, Gates Foundation, USA
- Dr Mark Alderson, PATH, USA

### Site PIs

- Dr Dean Everett, Malawi
- Dr Anne von Gottberg, South Africa
- Dr Martin Antonio, The Gambia
- Dr Jean-Marc Collard, Niger
- Prof Gerd Pluscke, Switzerland
- Dr Peter Hughes, Uganda
- Dr Sylvian Mermon, New Caledonia
- Dr Mohammed Timinouni, Marocco
- Dr Benoît Garin, Madagascar
- Dr Rob Breiman, Kenya
- Dr Jennifer Moïsi, Burkino Faso
- Dr Lesley McGee, USA
- Prof Amanda Leach & Dr Heidi Smith Vaughan, Australia

### Postdoctoral Scientists

- Dr Simon R. Harris
- Dr Jennifer Cornick

### MSc Scholarships

- Shanil Govindpershad, South Africa
- Madikay Senghore, The Gambia
- Chrispin Chaguza, Malawi

### Site Bioinformaticians

- Shanil Govindpershad
- Madikay Senghore
- Chrispin Chaguza

### Bioinformatics Support and Development

- Feyruz Yalcin

### Administrative Team

- Esther Chirombo (Program Manager)
- Julie Clark (Finance)
- Kate Jones, WTTC (Oversight)
